# *FLT3‐ITD* Measurable Residual Disease in Acute Myeloid Leukemia: Recommended, but Not Yet Self‐Sufficient

**DOI:** 10.1002/hon.70236

**Published:** 2026-08-04

**Authors:** Francesco Albano

**Affiliations:** ^1^ Department of Precision and Regenerative Medicine and Ionian Area (DiMePRe‐J)‐Hematology and Stem Cell Transplantation Unit University of Bari “Aldo Moro” Bari Italy

**Keywords:** acute myeloid leukemia, *FLT3*, measurable residual disease, PCR‐NGS, prognosis

## 1 Introduction

The history of FMS‐like tyrosine kinase 3‐internal tandem duplication (*FLT3‐ITD*) in acute myeloid leukemia (AML) is that of a molecular marker that is biologically powerful, clinically influential, but methodologically irregular. *FLT3‐ITD* has been associated with an increased risk of relapse and adverse prognosis, becoming a central element in the molecular risk stratification of AML. The development of *FLT3* inhibitors has further strengthened the clinical relevance of this marker, transforming *FLT3‐ITD* from a negative prognostic factor into a therapeutic target [1]. Against this background, the use of *FLT3‐ITD* as a measurable residual disease (MRD) marker is conceptually attractive: a frequent, prognostically relevant, and pharmacologically targetable lesion could enable early identification of persistent disease and guide targeted therapeutic interventions. However, the trajectory of *FLT3‐ITD* as an MRD marker has been less straightforward than that of other molecular targets in AML, such as mutated *NPM1* or fusion transcripts in core‐binding factor leukemias [2]. *FLT3‐ITD* is not a recurrent point mutation, but rather a heterogeneous family of insertional events that vary in number, mutational burden, length, insertion site, and sequence. This architecture has historically made the development of robust and generalizable MRD assays difficult. Beyond these technical constraints, substantial biological limitations must also be considered. *FLT3‐ITD* is often a signaling lesion acquired relatively late in the leukemic clonal hierarchy, frequently preceded by founder or preleukemic events [3]. It may therefore be subclonal, multiclonal, unstable, acquired at relapse, or selectively suppressed by *FLT3* inhibitor therapy [4, 5]. In recent years, technical advances have reopened the possibility of using *FLT3‐ITD* as a target for MRD monitoring. Dedicated polymerase chain reaction–next‐generation sequencing (PCR‐NGS) approaches can longitudinally track ITD at very low burden by combining targeted amplification, deep sequencing, and dedicated bioinformatic pipelines [6, 7]. Persistence of *FLT3‐ITD* detected by NGS‐based methods during remission is associated with an increased risk of relapse and death, particularly in the pre‐hematopoietic cell transplant (HCT) and peri‐HCT settings [8, 9, 10, 11, 12, 13]. Moreover, recent data suggest that, in selected contexts, MRD results may help inform *FLT3*‐directed strategies, such as post‐HCT maintenance with gilteritinib [14]. This evolving evidence has been incorporated into recent ELN‐DAVID recommendations, which now recognize a role for *FLT3‐ITD* ultra‐high‐sensitivity NGS MRD in selected clinical settings [2, 15]. Nevertheless, the transition from measurability to full clinical actionability remains complex. The ability to detect *FLT3‐ITD* at extremely low levels does not automatically imply that every signal is biologically representative, clinically timely, or therapeutically decisive. This Perspective stems from that ambivalence: *FLT3‐ITD* PCR‐NGS is now a technically powerful tool and is clinically recommended in specific windows, but it is not yet self‐sufficient as a universal platform for MRD surveillance. Its value depends on assay quality, the stability of the tracked clone, the timing of sampling, and the existence of a therapeutic decision genuinely linked to the result.

### 1.1 What PCR‐NGS Adds—And What It Requires

The reason why PCR‐NGS has reopened the *FLT3‐ITD* MRD question is strictly technical: it allows a heterogeneous insertional lesion, located in the juxtamembrane domain of *FLT3* and difficult to monitor with conventional fragment analysis, to be converted into a patient‐specific molecular signature that can potentially be tracked over time. This enables longitudinal detection of the ITD during remission, even at very low burden, requiring an architecture different from that of an ordinary diagnostic NGS panel. The assay must be designed to maximize sensitivity for a complex mutational event, while managing insertion length, variable breakpoints, amplification bias, multiclonality, and background noise. At these levels, raw read depth is not sufficient: real sensitivity depends on DNA input, the number of informative molecules, error control, sample quality, and a dedicated bioinformatic pipeline [15]. Bioinformatics is an integral component of the assay. Generic variant callers may be insufficient for *FLT3‐ITD*, because the event to be identified is not a nucleotide substitution but an internal duplication with variable breakpoints [7]. Dedicated algorithms are therefore required to reconstruct the ITD sequence, length, position, and relative abundance, and to longitudinally compare the diagnostic clone with the one detected during remission or at relapse [7]. However, dependence on assay‐specific pipelines introduces a problem of comparability across laboratories, thresholds, and platforms [2, 15]. The discussion of *FLT3‐ITD* PCR‐NGS cannot therefore be reduced to the declared analytical sensitivity. A sensitivity in the range of 10^−4^ or 10^−5^ is credible only if the sample contains a sufficient number of truly interrogable cell equivalents. In post‐chemotherapy or post‐HCT samples, low cellularity, DNA quality, and hemodilution may profoundly affect the effective limit of detection. The recent ELN‐DAVID update recommends a DNA input of no less than 500 ng, with 700 ng representing the ideal amount to ensure optimal sensitivity of the *FLT3‐ITD* NGS‐MRD assay [15]. Finally, working at extremely low variant allele frequency (VAF) increases the importance of quality controls. Contamination, index hopping, carry‐over from high‐burden samples, PCR errors, and background noise may generate apparently relevant signals. At the ultra‐low VAF levels targeted by *FLT3‐ITD* PCR‐NGS MRD, the clinical meaning of a positive result depends not only on whether a signal is detected, but also on whether it can be quantified reliably. This is why the distinction between limit of detection (LOD) and limit of quantification (LOQ) is not merely analytical, but clinically relevant [2]. A result that is “detectable below LOQ” should therefore be reported as a distinct category and should not be overinterpreted as a robust quantitative marker of disease burden, especially when it may influence a therapeutic decision [2, 9]. Reporting is part of test quality. A *FLT3‐ITD* PCR‐NGS report should not be limited to “positive” or “negative”, but should include LOD, LOQ, clonal architecture, VAF trends, diagnostic correspondence, and sample limitations. Thus, PCR‐NGS has certainly made *FLT3‐ITD* detectable as an MRD marker, but it has not made the test automatically simple or universally transferable: sensitivity, quantification, sample quality, error control, clonal tracking, and reporting must be validated as inseparable parts of the same process.

### 1.2 Clinical Evidence and Actionable Windows

Evidence accumulated over the past few years indicates that molecular persistence of *FLT3‐ITD* detected by PCR‐NGS is not merely an analytical epiphenomenon. Across several cohorts, positivity during morphologic remission is associated with a higher risk of relapse and inferior survival [8, 9, 10, 11, 12, 13, 14, 16]. The critical point, however, is to distinguish three levels: prognostic value, which is now well supported; clinical actionability, which is more convincing in peri‐HCT settings; and generalized routine use, which has not yet been fully demonstrated. The pre‐HCT setting currently represents the most robust context for *FLT3‐ITD* MRD assessment by PCR‐NGS. Allo‐HCT is one of the main post‐remission strategies for patients with *FLT3‐ITD* AML, but post‐HCT relapse remains frequent. Detecting molecular persistence before conditioning, at levels not captured by conventional methods, may therefore refine risk stratification [8, 9, 10]. In the pre‐HCT setting, studies including Pre‐MEASURE and subsequent *FLT3*‐focused analyses showed that residual *FLT3‐ITD*, particularly at VAF ≥ 0.01%, refines relapse risk and post‐HCT outcome [8, 9]. These data support the use of the test as a pre‐HCT risk assessment tool, although they do not prove that every result‐guided intervention improves outcome. The peri‐HCT and early post‐HCT phases represent the contexts in which *FLT3‐ITD* MRD comes closest to clinical actionability, because the result may inform a concrete therapeutic decision: maintenance or pre‐emptive intervention with a *FLT3* inhibitor. The MRD analysis of the MORPHO trial showed that the benefit of gilteritinib appeared to be concentrated in patients with detectable *FLT3‐ITD* MRD during the peri‐HCT period, whereas it was less evident in MRD‐negative patients [16]. Consistently, Jiang et al. [14] showed that *FLT3‐ITD* positivity at day +30 post‐HCT, assessed by PCR‐NGS on bone marrow, was associated with a higher risk of subsequent relapse or multiparameter flow cytometry (MFC)‐MRD and may contribute to the selection of patients eligible for *FLT3* inhibitor maintenance. Outside the transplant setting, AMLSG16‐10 and QuANTUM‐First indicate that *FLT3‐ITD* MRD clearance or persistence after intensive therapy correlates with relapse and survival [12, 13]. However, it has not been established whether residual positivity after induction or consolidation should systematically modify therapy outside a clinical protocol. For patients not proceeding to allo‐HCT because of age, comorbidity, or personal preference, serial *FLT3‐ITD* PCR‐NGS monitoring may nevertheless become clinically informative. In principle, molecular persistence or re‐emergence during lower‐intensity therapy or maintenance could prompt closer reassessment, integration with *NPM1* and MFC, or enrollment in trials testing MRD‐guided initiation or modification of *FLT3* inhibition. Molecular‐failure studies provide proof of principle that *FLT3*‐directed treatment can induce molecular responses, although isolated *FLT3‐ITD* was not the trigger in most cases [17]. At present, however, no *FLT3‐ITD* threshold, sampling schedule, or intervention has been prospectively validated to improve outcome in this population [2, 15]. Therefore, such use should remain investigational and multiparametric, because *FLT3‐ITD* clearance may reflect selective clonal suppression rather than global leukemia eradication. Overall, clinical evidence supports *FLT3‐ITD* PCR‐NGS as a prognostic biomarker and, around HCT, as a potentially actionable tool. It does not yet support its isolated use as a universal MRD surveillance platform in all patients with *FLT3‐ITD* AML.

### 1.3 Biological Fragility and Limits of Self‐Sufficiency

**FIGURE 1:**
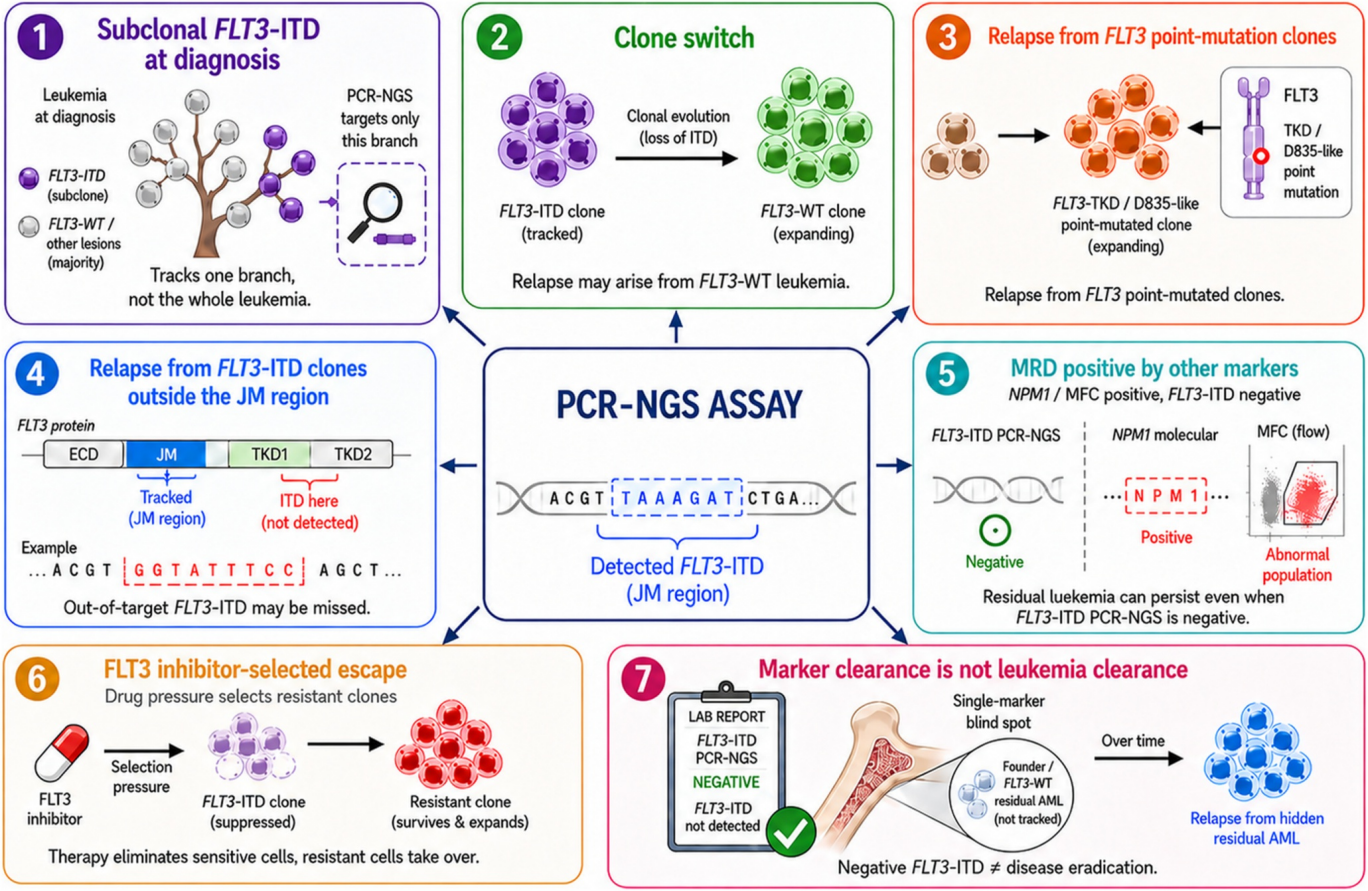
Biological mechanisms by which *FLT3‐ITD* PCR‐NGS MRD may miss or underestimate relapsing AML. The central panel illustrates a PCR‐NGS assay designed to detect *FLT3‐ITD* clone, usually within the juxtamembrane region. (1) *FLT3‐ITD* may be subclonal at diagnosis; therefore, the assay may follow only one leukemic branch rather than the whole disease. (2) During clonal evolution, the tracked *FLT3‐ITD* clone may be lost, while a *FLT3*‐WT clone expands and drives relapse. (3) Relapse may arise from cells carrying *FLT3* point mutations, such as TKD/D835‐like variants, which are not captured by an ITD‐focused assay. (4) *FLT3‐ITD* clones located outside the targeted juxtamembrane region may escape detection if the assay does not cover the relevant insertion site. (5) Residual disease may remain detectable by other MRD markers, such as *NPM1* or multiparameter flow cytometry, despite *FLT3‐ITD* PCR‐NGS negativity. (6) Under *FLT3* inhibitor pressure, sensitive *FLT3‐ITD*–positive cells may be suppressed, whereas resistant or less *FLT3*‐dependent clones survive and expand. (7) Finally, clearance of the *FLT3‐ITD* marker does not necessarily mean clearance of AML: a founder or *FLT3*‐WT residual population may persist below the scope of the assay and later re‐emerge. Overall, a negative *FLT3‐ITD* PCR‐NGS result should not be interpreted as proof of leukemia eradication, but as the absence of the tracked *FLT3‐ITD* signal in the tested sample. This figure was created with the assistance of the image‐generation functionality in ChatGPT (OpenAI), based on the author's original conceptual design and instructions. The author reviewed and edited the resulting images and assumes full responsibility for their scientific content and factual accuracy.

**FIGURE 2:**
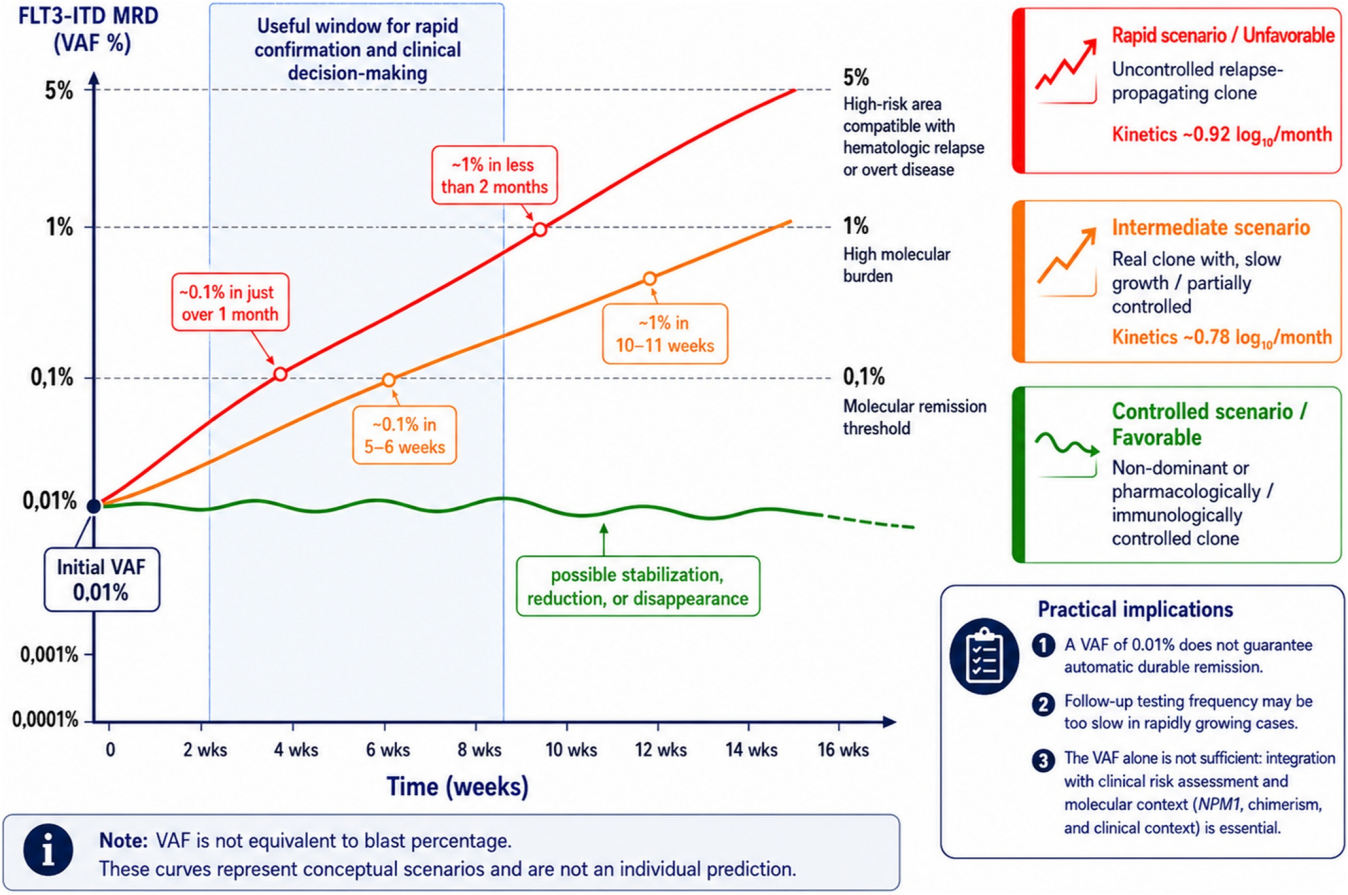
Conceptual modeling of *FLT3‐ITD* MRD trajectories from an ultra‐low positive threshold. The figure illustrates three hypothetical kinetic trajectories of *FLT3‐ITD* MRD, starting from an initial *FLT3‐ITD* VAF of 0.01%. The curves are intended as illustrative models and should not be interpreted as patient‐level predictions. The two rising curves were generated using a log‐linear growth model: VAF(t) = VAF_0_ × 10^(*k* × t) where VAF0 is 0.01%, k is the monthly log_10_ increase, and t is time in months. The rapid/unfavorable trajectory uses a growth constant of 0.92 log_10_/month, corresponding to the median relapse kinetics observed in patients with *FLT3‐ITD* mutated AML within a high‐risk AML cohort monitored by serial bone marrow flow cytometric MRD, as previously described (see ref. 20). The intermediate trajectory uses a growth constant of 0.78 log_10_/month, corresponding to the median relapse kinetics of the overall high‐risk AML cohort in the same study. Applied to an initial VAF of 0.01%, these kinetics predict an increase to approximately 0.1% in 4–6 weeks and to approximately 1% in 9–11 weeks, depending on the assumed growth rate. The controlled/favorable trajectory represents a biologically distinct scenario rather than a mathematically fitted growth model. In this case, the same low‐level *FLT3‐ITD* signal may remain stable, decline, or disappear if the detected clone is not relapse‐propagating, is pharmacologically suppressed by *FLT3* inhibition, or is controlled by post‐transplant graft‐versus‐leukemia effects. This scenario is supported by clinical observations showing that *FLT3‐ITD* MRD kinetics may diverge according to treatment exposure, clonal architecture, and post‐transplant immune control. The clinical message of the model is that an *FLT3‐ITD* VAF of 0.01% should not be regarded as automatically reassuring. If the detected clone follows a rapid signaling‐lesion relapse trajectory, the actionable window for confirmation, repeat sampling, integration with multiparameter flow cytometry, *NPM1* MRD, chimerism and clinical context, and initiation of a pre‐emptive *FLT3*‐directed intervention may be measured in weeks rather than months. Conversely, because *FLT3‐ITD* can be subclonal, unstable, treatment‐suppressed, or lost at relapse, a single ultra‐low positive result is insufficient to establish an inevitable clinical trajectory. This figure was created with the assistance of the image‐generation functionality in ChatGPT (OpenAI), based on the author's original conceptual design and instructions. The author reviewed and edited the resulting images and assumes full responsibility for their scientific content and factual accuracy.

The main limitation is not merely technical. *FLT3‐ITD* is not always a stable, founding, and fully representative marker of the entire MRD burden. PCR‐NGS can detect the persistence of a *FLT3‐ITD*‐positive clone with high sensitivity, but it does not guarantee that this clone corresponds to the leukemic population capable of sustaining relapse (Figure 1). *FLT3‐ITD* is often a signaling lesion acquired late in the clonal hierarchy, frequently preceded by founder or preleukemic events [3]. It may therefore reside on one or more subclonal branches rather than on the trunk of the leukemic tree. In this scenario, its detection describes a specific clonal trajectory, not necessarily the global persistence of AML; conversely, *FLT3‐ITD* negativity does not equate to molecular eradication of the disease. Clonal instability further limits the self‐sufficiency of *FLT3‐ITD* MRD. *FLT3‐ITD* may be lost at relapse, acquired during progression, or functionally replaced by alternative signaling pathways. This limitation is particularly relevant in the era of *FLT3* inhibitors: the selective pressure exerted by midostaurin, quizartinib, gilteritinib, or sorafenib may reduce the *FLT3*‐dependent compartment without eliminating the entire leukemic architecture [4, 5, 11]. Clearance of *FLT3‐ITD* MRD during treatment should therefore be interpreted as suppression of the tracked clone, not as proof of global AML eradication. Multiclonality amplifies this uncertainty. Multiple *FLT3‐ITD* clones may coexist in the same patient, differing in insertion length, insertion site, VAF, therapeutic sensitivity, and evolutionary fate [18]. More broadly, alternative *FLT3*‐altered clones, including point‐mutated clones, may coexist or emerge [4]. A clone that is dominant at diagnosis may disappear, whereas a minor clone may persist or expand; even summed VAFs may be misleading because they aggregate biologically distinct trajectories. In fact, VAF is not a direct measure of blast percentage or leukemic mass, and should be interpreted as a trajectory integrating level, change over time, sample source, and therapeutic context. Proliferative kinetics represent an additional operational limitation. Signaling mutations may be associated with a short interval from molecular detection to hematologic relapse [19]. MFC‐MRD surveillance data in high‐risk AML indicate a median pre‐relapse growth rate of 0.78 log_10_/month, reaching 0.92 log_10_/month in patients with *FLT3* mutations at diagnosis [20]. These data do not describe serial *FLT3‐ITD* VAF growth by PCR‐NGS, but support an illustrative model of compressed MRD‐to‐relapse kinetics in *FLT3*‐mutated AML (Figure 2), not an individualized prediction of relapse. Nevertheless, the model clarifies a practical point: PCR‐NGS positivity at or above 0.01% should not be regarded as a marginal signal to be reassessed after a long interval, but as a molecular alert requiring rapid confirmation and interpretation according to serial kinetics. The practical consequence is that *FLT3‐ITD* MRD should not be used as the sole measure of residual disease. *FLT3‐ITD* negativity is truly reassuring only when it is consistent with MFC, stable molecular markers, post‐HCT chimerism, clinical context, and sample quality. In patients with *NPM1*‐mutated/*FLT3‐ITD* AML, *NPM1* may serve as a more stable marker of global leukemic persistence, whereas *FLT3‐ITD* provides additional information on the persistence of a high‐risk, proliferative, and pharmacologically targetable branch. The interpretation of an ultra‐low *FLT3‐ITD* detection should also be contextualized within the baseline co‐mutational architecture, although current evidence does not support genotype‐specific thresholds for *FLT3‐ITD* MRD. In *NPM1*‐mutated AML, concordant persistence of *NPM1* and *FLT3‐ITD* is biologically more consistent with persistence of the diagnostic leukemic population, whereas isolated *FLT3‐ITD* positivity or clearance requires greater caution because *FLT3‐ITD* may represent a later subclone. Conversely, mutations associated with clonal hematopoiesis, such as *DNMT3A*, *TET2*, and *ASXL1*, may persist during remission without themselves indicating residual AML, but identify an ancestral substrate from which *FLT3*‐independent relapse may emerge. Adverse‐risk lesions, including *RUNX1*, *TP53*, or *WT1*, may further shape relapse risk and limit the reassurance provided by clearance of the *FLT3‐ITD* signal [21]. Thus, co‐mutations should contextualize, rather than independently redefine, the PCR‐NGS result, and their combined value requires prospective validation. Therefore, *FLT3‐ITD* PCR‐NGS is best understood as a dynamic biomarker of risk, clonality, and therapeutic vulnerability, rather than as a universal, self‐sufficient indicator of MRD.

## 2 Conclusions

*FLT3‐ITD* PCR‐NGS has transformed a historically difficult‐to‐track marker into a molecular signal that can be monitored at previously unrealistic depths. Available evidence shows that persistence of *FLT3‐ITD* during remission, particularly in the pre‐HCT and peri‐HCT settings, identifies patients at increased risk of relapse and death. In selected contexts, such as post‐HCT maintenance with gilteritinib, this information may also help identify patients potentially eligible for *FLT3*‐directed intervention. However, analytical maturity does not equate to clinical self‐sufficiency. *FLT3‐ITD* PCR‐NGS should be considered not as a universal and autonomous MRD surveillance platform, but as a dynamic biomarker of risk, clonality, and potential actionability. Its most convincing space is selective: pre‐HCT risk stratification, peri‐HCT and early post‐HCT assessment, support for decisions regarding maintenance or *FLT3*‐directed strategies, and development of MRD‐guided trials. Until the chain linking marker, method, timing, interpretation, and intervention is robustly defined and standardized, routine implementation should remain prudent and selective.

## Funding

The author has nothing to report.

## Ethics Statement

The author has nothing to report.

## Consent

The author has nothing to report.

## Conflicts of Interest

The author declares no conflicts of interest.

## Permission to Reproduce Material From Other Sources

The author has nothing to report.

## Data Availability

The author has nothing to report.
